# Examination of an eHealth literacy scale and a health literacy scale in a population with moderate to high cardiovascular risk: Rasch analyses

**DOI:** 10.1371/journal.pone.0175372

**Published:** 2017-04-27

**Authors:** Sarah S. Richtering, Rebecca Morris, Sze-Ee Soh, Anna Barker, Fiona Bampi, Lis Neubeck, Genevieve Coorey, John Mulley, John Chalmers, Tim Usherwood, David Peiris, Clara K. Chow, Julie Redfern

**Affiliations:** 1 The George Institute for Global Health, Sydney, NSW, Australia; 2 Hôpitaux Universitaires de Genève, Université de Genève, Geneva, Switzerland; 3 School of Public Health and Preventive Medicine, Faculty of Medicine, Nursing and Health Sciences, Monash University, Melbourne, Victoria, Australia; 4 Department of Physiotherapy, Monash University, Melbourne, Victoria, Australia; 5 Sydney Nursing School, Charles Perkin Centre, University of Sydney, Sydney, NSW, Australia; 6 School of Health and Social Care, Edinburgh Napier University, Edinburgh, Scotland, United Kingdom; 7 Sydney Medical School, University of Sydney, NSW, Sydney, Australia; 8 Westmead Hospital, Sydney, NSW, Sydney, Australia; University Of São Paulo, BRAZIL

## Abstract

**Introduction:**

Electronic health (eHealth) strategies are evolving making it important to have valid scales to assess eHealth and health literacy. Item response theory methods, such as the Rasch measurement model, are increasingly used for the psychometric evaluation of scales. This paper aims to examine the internal construct validity of an eHealth and health literacy scale using Rasch analysis in a population with moderate to high cardiovascular disease risk.

**Methods:**

The first 397 participants of the CONNECT study completed the electronic health Literacy Scale (eHEALS) and the Health Literacy Questionnaire (HLQ). Overall Rasch model fit as well as five key psychometric properties were analysed: unidimensionality, response thresholds, targeting, differential item functioning and internal consistency.

**Results:**

The eHEALS had good overall model fit (χ^2^ = 54.8, p = 0.06), ordered response thresholds, reasonable targeting and good internal consistency (person separation index (PSI) 0.90). It did, however, appear to measure two constructs of eHealth literacy. The HLQ subscales (except subscale 5) did not fit the Rasch model (χ^2^: 18.18–60.60, p: 0.00–0.58) and had suboptimal targeting for most subscales. Subscales 6 to 9 displayed disordered thresholds indicating participants had difficulty distinguishing between response options. All subscales did, nonetheless, demonstrate moderate to good internal consistency (PSI: 0.62–0.82).

**Conclusion:**

Rasch analyses demonstrated that the eHEALS has good measures of internal construct validity although it appears to capture different aspects of eHealth literacy (e.g. using eHealth and understanding eHealth). Whilst further studies are required to confirm this finding, it may be necessary for these constructs of the eHEALS to be scored separately. The nine HLQ subscales were shown to measure a single construct of health literacy. However, participants’ scores may not represent their actual level of ability, as distinction between response categories was unclear for the last four subscales. Reducing the response categories of these subscales may improve the ability of the HLQ to distinguish between different levels of health literacy.

## Introduction

For patients to be able to optimally manage their health, they require an adequate level of understanding about their condition and associated management strategies [1–3]. This is related to an individual’s level of health literacy and is an increasingly important area of research particularly in people with chronic disease. Cardiovascular disease (CVD), for example, is a major health burden for patients and often requires life-long behaviour changes across multiple risk factors [4]. CVD management depends on active patient participation, which requires a sufficient level of health literacy [5]. It has been shown that patients with CVD that have a low level of health literacy are less likely to adhere to prescribed medications [4–6]. Similarly, lack of adherence to prescribed medication has also been found to be associated with inadequate or marginal health literacy [4].

Electronic health (eHealth) literacy has become increasingly relevant in recent years with the development of eHealth tools to support healthcare delivery and management [7–9]. However, with these innovative developments eHealth literacy becomes an equally important area of investigation. eHealth literacy is defined as “the ability to seek, find, understand, and appraise health information from electronic sources and apply the knowledge gained to addressing or solving a health problem” [10]. The additional challenge of eHealth relates to the skills required to use an electronic device as well as having an adequate level of health literacy to effectively make decisions regarding health [2,9]. In order to ensure the existing scales for health and eHealth literacy are clinically applicable, it therefore becomes important to examine how they perform in various populations when developing, evaluating and implementing health management strategies.

Several scales can be used to measure health literacy such as the Test of Functional Health Literacy in Adults (TOFHLA) [11] and the Rapid Estimate of Adult Literacy in Medicine (REALM) [12]. However, these scales assess different areas of health literacy which means that an individual can have a very different level of health literacy depending on the scale used [13]. A more comprehensive measure of health literacy is the Health Literacy Questionnaire (HLQ) [14]. In contrast, there are currently very few scales available to evaluate eHealth literacy [7]. One such scale is the electronic Health Literacy Scale (eHEALS) [15]. The psychometric properties of both the eHEALS and the HLQ have been examined using classical test theory and item response theory methods. Prior studies using confirmatory factor analysis (CFA) have demonstrated the eHEALS to be a valid and reliable scale [16,17]. More recent item response theory studies have confirmed these findings [18,19] and demonstrated that the scale measures the same underlying concept or construct. However, a recent study found that the eHEALS was not able to capture the full range of eHealth literacy levels in their study population (i.e. ceiling and floor effects) [18]. The factor structure of the HLQ has also been tested with CFA and three subscales tested were found to be measuring the same underlying construct [19]. It has also demonstrated satisfactory reliability [14]. However discrimination between response categories was questioned for three of the nine scales [14,20], suggesting that response categories could be revised to improve the scales measurement properties.

There is growing evidence to indicate that the Rasch measurement model, which measures a scale’s internal construct validity, is the gold standard for psychometric evaluations of outcome scales [21]. Rasch analysis has several well-recognised advantages over classical test theory including analysis of item summation legitimacy, response category distinction, hierarchical structure of item difficulty and discrepancies in item response for a given level of ability [22,23], which all strengthen scale construct validation. Having valid scales is particularly relevant in a population at risk for CVD where an adequate level of health literacy has been shown to improve effective patient disease management [24]. Moreover, there has not yet been a Rasch validation study in a population with CVD. Given that CFA may not fully resolve issues associated with the conceptual structures of psychological scales such as health literacy [20], further evaluation using the Rasch measurement model is required to extend the current knowledge on the validity of these scales. The aim of this study was to extend prior validation studies by examining the internal construct validity of an eHealth and a health literacy scale using Rasch analysis to provide clinicians and researchers with information on the usefulness of these scales.

## Materials and methods

### Design

The internal construct validity of the eHEALS and the HLQ was analysed using Rasch analysis in a population with moderate to high CVD risk. The first 397 consented participants in the CONNECT (Consumer Navigation of Electronic Cardiovascular Tools) Study [25] comprised the sample and completed the eHEALS and HLQ scales. All participants provided written, informed consent and ethical approval was obtained from the University of Sydney Human Research Ethics Committee (Project number 2013/091).

### Participants and setting

Details of the CONNECT Study have been published elsewhere [25]. In brief, it is a randomised controlled trial examining whether an eHealth strategy improves risk factor control when compared with usual health care in patients at risk of or with CVD. Participants were recruited via Australian primary care practices. To be eligible, they had to be 18 years or older, have access to the Internet at least once a month (mobile phone, tablet or computer) and have moderate to high risk for CVD. Moderate to high CVD risk was defined as (a) ≥ 10% 5-year CVD risk using the Framingham risk equation; (b) a clinically high risk condition (Aboriginal or Torres Strait Islander > 75 years, diabetes and >60 years, diabetes and albuminuria, estimated glomerular filtration rate < 45 ml/min, systolic blood pressure ≥180 mmHg, diastolic blood pressure ≥110 mmHg, total cholesterol > 7.5 millimol); (c) an established CVD diagnosis (ischaemic heart disease, stroke/transient ischaemic attack, peripheral vascular disease). Participants with an insufficient level of English to provide informed consent or severe intellectual disability were excluded.

### Scales used for assessment of eHealth literacy and general health literacy

The eHEALS was used to assess eHealth literacy (Study 1). This scale aims to measure an individual’s perception of their knowledge and skills in relation to using electronic health information and determine whether an eHealth approach is suited to the individual [15,26]. It is an 8-item scale with each item scored on a 5-point Likert scale (Table 1). The sum across the eight equally weighted items is presented as a score out of 40. There is no fixed cut-off to distinguish high from low eHealth literacy but higher scores reflect a higher level of eHealth literacy [15,27]. The scale was completed online directly by participants themselves.

**Table 1:** Description of the eHEALS (electronic Health Literacy Scale) items and HLQ (Health Literacy Questionnaire) subscales.

**eHEALS**	
Item 1^a^	I know what health resources are available on the Internet
Item 2^a^	I know where to find helpful health resources on the Internet
Item 3^a^	I know how to find helpful health resources on the Internet
Item 4^a^	I know how to use the Internet to answer my questions about health
Item 5^a^	I know how to use the health information I find on the Internet to help me
Item 6^a^	I have the skills I need to evaluate the health resources I find on the Internet
Item 7^a^	I can tell high quality health resources from low quality health resources on the Internet
Item 8^a^	I feel confident in using information from the Internet to make health decisions
**HLQ**	
Subscale 1^b^	Feeling understood and supported by healthcare provider (4 items)
Subscale 2^b^	Having sufficient information to manage my health (4 items)
Subscale 3^b^	Actively managing my health (5 items)
Subscale 4^b^	Social Support for health (5 items)
Subscale 5^b^	Appraisal of health information (5 items)
Subscale 6^c^	Ability to actively engage with healthcare providers (5 items)
Subscale 7^c^	Navigating the healthcare system (6 items)
Subscale 8^c^	Ability to find good health information (5 items)
Subscale 9^c^	Understanding health information well enough to know what to do (5 items)

^a^Response categories: strongly disagree, disagree, undecided, agree, and strongly agree.

^b^Response categories: strongly disagree, disagree, agree, and strongly agree.

^c^Response categories: cannot do, very difficult, quite difficult, quite easy and very easy.

The HLQ was used to assess health literacy (Study 2). This scale aims to measure an individual’s capacity to effectively use health information and services [14]. It is a 44-item questionnaire with 9 subscales (Table 1). Subscales 1 to 5 are scored on a 4-point Likert scale while the subscales 6 to 9 are 5-point Likert scale. The score for the items in each subscale is summed and divided by the number of items providing nine individual scores. There is no total score across subscales. Although there are no fixed values to classify the level of health literacy, similar to the eHEALS, higher scores indicate higher health literacy in all subscales [14,28].

### Model used to assess psychometric properties

The Rasch model was used to examine the psychometric properties of the eHEALS and HLQ. Rasch analysis is a form of item response theory, where the ordinal ratings of the questionnaire are transformed to estimates of interval measures that demonstrate the essential features of the scale [23]. Analysis with the Rasch model provides difficulty measures for each item and ability estimates for each participant located on the same measurement scale as a log of the odds units, or logits. This allows expected and observed results to be compared and the internal construct validity of each item to be determined [23]. This will determine how well the items and participants fit the Rasch model i.e. overall model fit. We also examined five key psychometric parameters detailed in Table 2. To ensure an appropriate degree of precision from the Rasch analysis, a minimum sample size between 108 to 243 participants is required [29].

**Table 2:** Description of the five key psychometric parameters assessed in a Rasch analysis to test the psychometric properties of a scale.

Parameter	Definition/Aim	Measurement
**Unidimensionality**	The extent to which the items of a scale measure a single construct (or concept). All items must measure a single construct for them to be summed.	Subsets of items were defined by positive and negative loadings on the first factor extracted using a principal component analysis of residuals [21,23,30]. Independent t-tests were then used to compare person estimates derived from the two most dissimilar subsets of scale items. The scale was unidimensional if independent t-test <0.05 i.e. less than 5% show a significant difference between their scores on the two subsets. If t-test >0.05, the value of 5% should fall within the 95% CI around the t-test estimate calculated with a binomial test of proportions [23,31]
**Response Thresholds**	Reflects the distance between response categories to determine whether participants had difficulty discriminating between them	Category probability curves [23] were used to identify the presence of disordered thresholds and attempts to order them were made by collapsing response categories. Response categories were deemed ordered when each response systematically had a point along the location/ability continuum where it was the most likely response (indicated by a peak in the curve)
**Targeting**	Representation of the extent to which the spread of items reflects the levels of ability (e.g. health literacy) within the sample.	Person-item threshold distribution maps, which reflect the mean location score obtained for the persons with that of the value of zero, [23] were analysed for (1) the presence of ceiling/floor effects i.e. whether extremes of ability were not accounted for by the scale, (2) to ensure that all mid-ranges of health literacy were also represented. A mean location for the persons would be around zero for a well-targeted scale. Positive mean value for the persons indicates that the sample is overqualified for the scale (e.g. have higher health literacy) and negative values, the opposite. [23]
**Differential Item Functioning (DIF)**	Demonstrates whether different groups with equal ability score a given item differently	Analysis of variance with a Bonferonni adjusted alpha level (p < 0.05/(2*items) [23]. Subgroups analysed: age (<64, 64–69, >69 years), gender (male or female), polypharmacy (active consumption of >4 or <4 medications), level of education (none/primary/secondary, university studies or technical/vocational training). Uniform DIF is indicated by a significant main effect for the person factor (e.g. age) and can be remedied by calibrating the item for each group [23]. Non-uniform DIF is indicated by a significant interaction effect and often indicates that there may be an issue around item fit [23]
**Person Separation Index (PSI)**	Reflects the internal consistency of the scale and the extent to which items distinguish between levels of health literacy (analogous to Crohnbach α)	PSI between 0.70–0.90 [23] with >0.90 indicating possible item redundancy

### Analysis

The CONNECT data were analysed using IBM SPSS Statistics 22.0 (IBM SPSS Statistics for Windows Armonk, NY: IBM Corp), with the Rasch analysis completed using the RUMM2030 package using a partial credit model for polytomous data (RUMM Laboratory Pty Ltd, Perth, Australia). Given that both the 8-item eHEALS and HLQ have multiple response category options (e.g. ‘strongly disagree’ to ‘strongly agree’), a partial credit Rasch model was used to examine the internal construct validity of both scales, which is not possible with, for example, a two-parameter logistic (2PL) model. [21] To determine whether the observed data fit the expectations of the Rasch model (overall model fit) the item-trait interaction statistic was used, which were reported as a χ^2^ statistic. A significant value (p<0.05) indicated that the observed data did not fit the expectations of the Rasch model [23]. It is important to keep in mind that the χ^2^ test is sensitive to sample size, with larger samples having a tendency to generate a significant value [32]. Model fit was also assessed by examining item-person interaction statistics, where a residual standard deviation (SD) of >1.5 suggested there may be an issue with fit [23,33] as well as residual fit statistics of individual item- and person-fit statistic where values > ± 2.5 [33] indicated misfitting items or persons. The details of the five key psychometric parameters assessed are described in Table 2 [27].

## Results

Demographic characteristics of the 397 consenting participants are presented in Table 3. Three-quarters of the sample were male, with a mean age of 66.3 years (SD 8.1), 88% were Caucasian and the majority (79%) were married or in a defacto relationship. Due to incomplete scale completion, one participant was removed from both the eHEALS and HLQ for the Rasch analysis. The mean total score for the eHEALS was 27.1 (range: 8–40; SD 6.67), 3.03 (range: 1–4; SD 0.52) for the first five HLQ subscales and 4.19 (range: 1–5; SD 0.47) for subscales 6 to 9 (Table 4).

**Table 3:** Demographic characteristics of cohort.

Variable		N = 397 (%)
**Male, n (%)**		304 (77)
**Age (years)**		
	<64, n (%)	122 (31)
	64–69, n (%)	149 (37)
	≥70, n (%)	126 (32)
**Ethnicity**		
	Caucasian, n (%)	353 (89)
	Non-Caucasian^a^, n (%)	44 (11)
**Relationship status**		
	Married/Defacto, n (%)	316 (80)
	Single/Divorced/Widowed, n (%)	80 (20)
	Missing, n (%)	1 (0.3%)
**Education qualification **		
	None/Primary/Secondary school, n (%)	108 (27)
	Undergraduate/Postgraduate degree or diploma, n (%)	208 (52)
	Technical/vocational qualification, n (%)	81 (20)
**Polypharmacy**^b^		165 (42)
**Home/work access to Internet**		392 (99)
**Income **		
	$ <1,000 per week, n (%)	109 (28)
	$ 1,000–2,000 per week, n (%)	126 (32)
	$ >2,000 per week, n (%)	113 (29)
	Participant chose not to answer, n (%)	49 (12)
**Private health insurance, n (%)**		322 (81)

^a^Aboriginal/Torres Strait Islander/Pacific Islander/South Asian/Other Asia/Middle East/Mediterranean/Other.

^b^Active consumption of >4 medications.

**Table 4:** Mean scores and overall Rasch model fit statistics, unidimensionality, thresholds and internal consistency of electronic Health Literacy Scale (HEALS) and Health Literacy Questionnaire (HLQ)^a^.

	Ideal	eHEALS	HLQ-1	HLQ-2	HLQ-3	HLQ-4	HLQ-5	HLQ-6	HLQ-7	HLQ-8	HLQ-9
Mean scores (±SD)	N/A	27.1 (6.67)	3.38 (0.45)	2.93 (0.47)	2.90 (0.48)	3.12 (0.48)	2.79 (0.53)	4.29 (0.52)	4.12 (0.55)	4.07 (0.56)	4.28 (0.47)
Total item-trait interaction											
Total item χ^2^		54.80	27.10	51.58	46.97	30.51	18.18	15.64	60.60	36.24	23.72
df		40	4	8	15	10	20	5	24	10	10
p-value	>0.05	0.06	0.00	0.00	0.00	0.00	0.58	0.01	0.00	0.00	0.01
Items											
Fit residual (mean)	0	-0.65	-2.8	-2.61	-2.58	-2.27	-0.9	-3.06	-1.72	-2.56	-2.58
Fit residual (SD)^c^	<1.5	2.31	0.68^b^	1.74	2.34 ^b^	2.19^b^	1.35^b^	0.79^b^	2..51^b^	0.63^b^	1.01^b^
Persons											
Fit residual (mean)	0	-0.81	-0.68	-0.74	-1.02	-0.94	-0.76	-0.96	-0.80	-0.89	-1.01
Fit residual (SD)^c^	<1.5	1.69	0.72	0.89	1.52^b^	1.43^b^	1.43^b^	1.15	1.19^b^	1.16	1.41
Unidimensionality											
% signification t-tests	<5%	12.60%	1.52%	3.54%	4.04%	3.79%	4.81%	5.05%	2.02%	3.79%	11.10%
(CI)	(lower limit <5%)	(0.11 to 0.15)	(-0.01 to 3.7)	(1.4 to 5.7)	(1.0 to 6.2)	(1.6 to 5.9)	(2.7 to 6.9)	(2.9 to 7.2)	(1.0 to 4.2)	(1.6 to 5.9)	(9.0 to 13.3)
Thresholds (Disordered items)	Ordered	Ordered	Ordered	Ordered	Ordered	Ordered	Ordered	Disordered (25)	Disordered (24, 34, 42)	Disordered (26, 29, 33, 41)	Disordered (28, 35, 40, 44)
Person-separation index^c^	>0.70	0.90	0.77	0.75	0.75	0.72	0.77	0.64	0.82	0.64	0.62

^a^As analysed using RUMM2030 (Rumm Laboratory Pty Ltd., Perth) for Windows.

^b^Contains individual item or person misfits and/or redundancies.

^c^Rasch based reliability statistic (analogous to Cronbach’s alpha).

SD, standard deviation; d*f*, degrees of freedom.

### Study 1: eHEALS

The eHEALS met the Rasch model expectations as demonstrated by the χ^2^ item-trait interaction statistic (p: 0.06) (Table 4). The scale did, however, indicate some degree of item misfit (fit residual mean -0.65, SD 2.31) and person misfit (fit residual mean -0.81; SD 1.69) as reflected in the item-interaction statistics [23]. Individual person-fit statistics also revealed several participants with fit residuals > ±2.5, which could be due to the scale’s limitation of detecting mid to high levels of eHealth literacy (see ‘Targeting’ lower down).

#### Unidimensionality

We found limited evidence to support unidimensionality of the eHEALS. Analysis using principal components analysis suggested that the eHEALS may be measuring two separate constructs of the eHealth literacy (p = 0.13; 95% CI 0.11, 0.15), which may indicate that items must be scored separately.

#### Response thresholds

Inspection of thresholds maps and category probability curves showed ordered thresholds for the five response categories (‘strongly disagree’ to ‘strongly agree’) used in the eHEALS scale, demonstrating that participants were able to distinguish between response options.

#### Targeting

**Fig 1:**
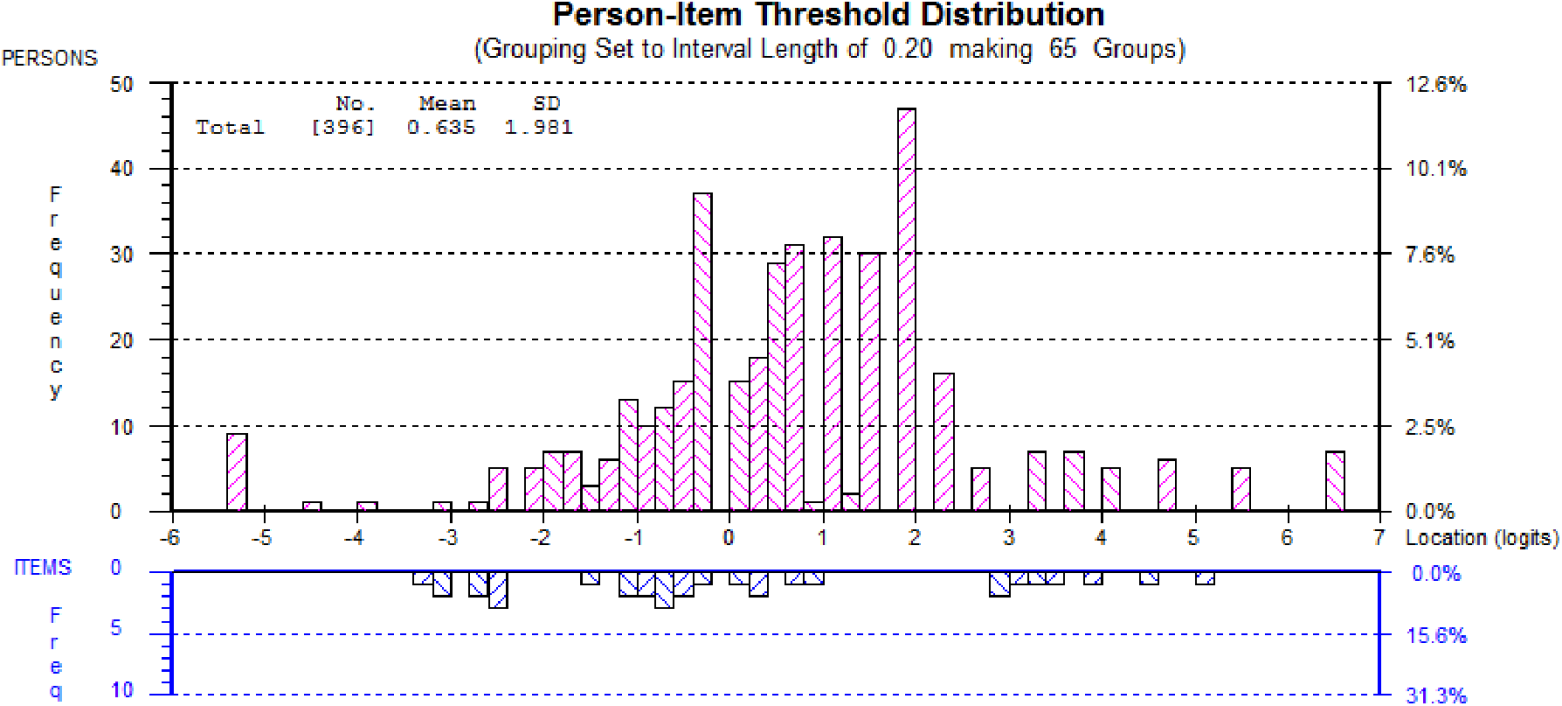
Targeting of the eHEALS as demonstrated by the person-item threshold distribution.

The eHEALS scale displayed reasonable targeting (Fig 1: overall spread of items on bottom half matched spread of persons in top half) with a mean logit score of 0.64 (N.B. ideal: 0). The scale was not, however, able to detect small but clinically important changes in participants with mid to higher levels of eHealth literacy (Fig 1: gap between logit 1 and 3 in the bottom half of the graph).

#### DIF (Item bias)

No significant DIF (or item bias) was evident in the eHEALS for age, gender, polypharmacy and education indicating no influence of these characteristics on response to any of the items of the scale.

#### PSI (internal consistency)

With a PSI of 0.90 (Table 4), the scale demonstrated good internal consistency.

### Study 2: HLQ

Only subscale 5 met the Rasch model expectations for good overall model fit (χ^2^ item-trait interaction statistic <0.05). The remaining HLQ subscales did not reflect hierarchical ordering of the items across all levels of health literacy (Table 4). Inspection of individual item-fit statistics revealed the presence of misfits and redundancies (S1 Table).

#### Unidimensionality

All subscales, apart from subscale 9 (p = 0.11; 95% CI 0.09, 0.13), displayed unidimensionality.

#### Response thresholds

**Fig 2:**
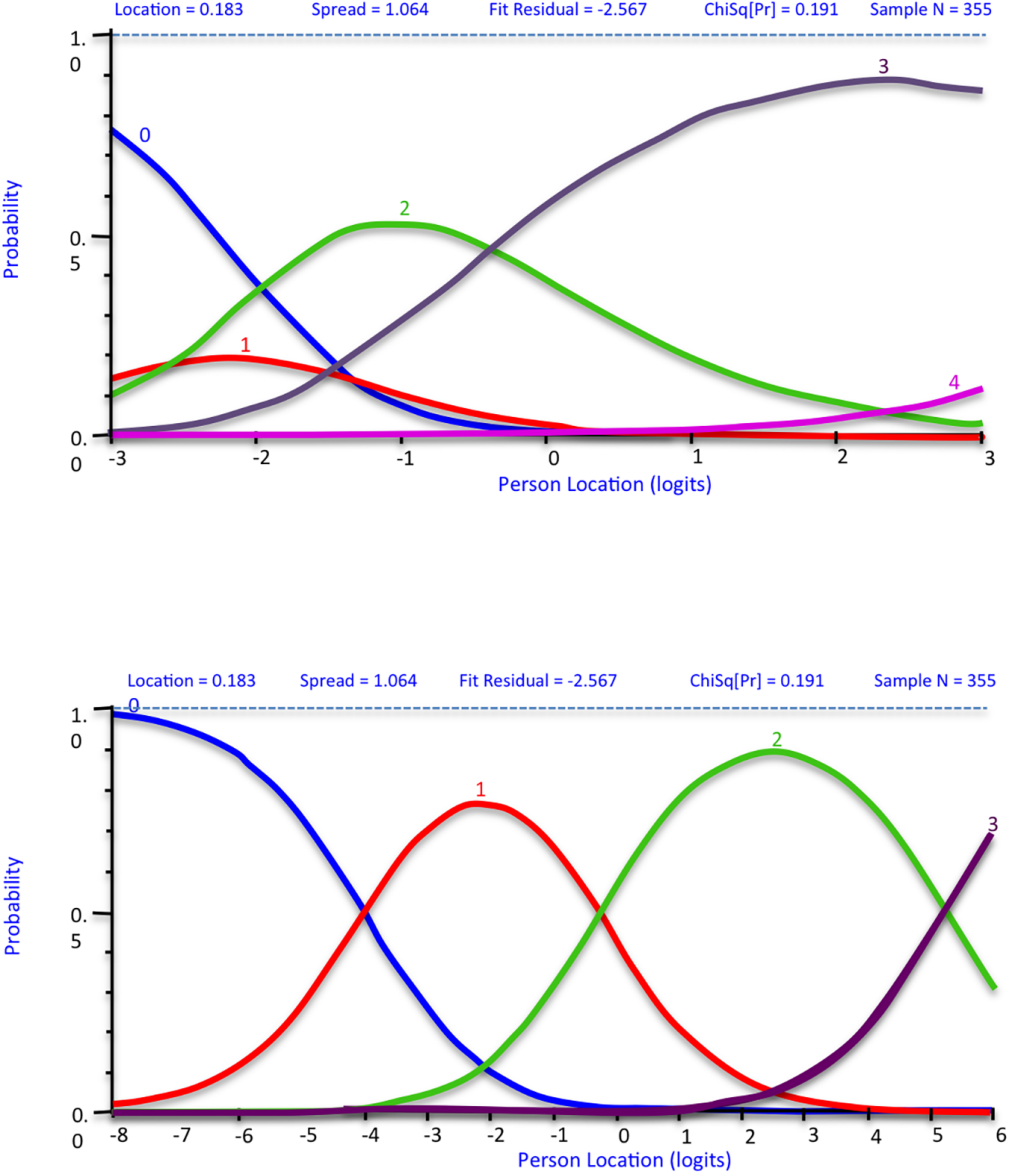
Category probability curve showing (a) disordered thresholds in HLQ subscale-9, Item 26 (b) ordered thresholds when response categories ‘very difficult’ and ‘quite difficult’ are collapsed into one.

Ordered thresholds were obtained for subscales 1 to 5 (‘strongly disagree’ to ‘strongly agree’). However, inspection of category probability curves for subscales 6 to 9 showed that participants had difficulty distinguishing between their response categories (‘cannot do’ to ‘quite easy’), as seen by the confluence of peaks in Fig 2A. When the response categories ‘very difficult’ and ‘quite difficult’ were collapsed into one category, response thresholds for these subscales became ordered (Fig 2B: distinct peaks for each response category).

#### Targeting

**Fig 3:**
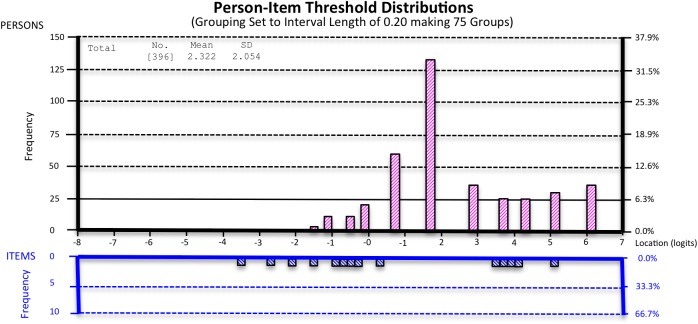
Targeting of the HLQ subscale-4 as demonstrated by the Person-item Threshold Distribution.

Targeting was suboptimal for all subscales of the HLQ (mean logit: 1.02 to 4.26) indicating the sample was slightly overqualified for the level of health literacy measured by the scale. This was further highlighted by the ceiling effect (Fig 3: absence of items in bottom half of graph for higher levels of health literacy represented on the top half). There were also gaps in the mid to high health literacy range.

#### DIF (Item bias)

Uniform DIF (p<0.05) was observed in HLQ subscales 2, 3, 4 and 8 for education, polypharmacy, gender and age, respectively. Subscale 3 displayed non-uniform DIF (p<0.05) for item 13 (“Despite other things in my life, I make time to be healthy”). However, in all cases, the DIF was not marked and isolated to one item per subscale with minor effect on each respective subscale.

#### PSI (internal consistency)

The PSI varied between 0.62 and 0.82 indicating moderate to good internal consistency for all nine HLQ subscales (Table 4).

## Discussion

This study found that the eHEALS had good overall fit to the Rasch model, distinct response categories, no item bias and reasonbale targeting. This provides evidence supporting the use of the 8-item eHEALS as a measure of eHealth literacy with higher scores truly representing higher levels of eHealth literacy. All subscales of the HLQ except for subscale 5, on the other hand, did not fit the Rasch measurement model and had suboptimal targeting. Nevertheless, all subscales apart from subscale 9 did demonstrate unidimensionality, had no major item bias and moderate to good internal consistency. This indicates that the HLQ suitably measures nine aspects of health literacy. Both scales were, however, unable to reflect mid to high ranges of health literacy. Consequently, when they are used to measure changes in ability over time, an individual would have to acquire a substantial increase in health or eHealth literacy for it to be reflected in their score. This could account for the presence of misfitting items and persons and highlights the potential for further scale testing and refinement.

Prior studies using factor analysis or principal component analysis (PCA) and item response theory (IRT) have found the eHEALS to be internally consistent (Crohnbach α: 0.87 to 0.94) with modest to good stability [7,16,17,34] and have supported the construct validity of the scale. The high internal consistency found in this study (PSI: 0.90) further supports these findings [7,16]. A recent study using IRT in a population with diverse chronic diseases demonstrated good model fit and distinct response categories for the eHEALS [19]. Good model fit was also found in another IRT study (in a student population and adults who use the Internet) with similar findings around targeting, notably a marked ceiling effect [18]. Unlike the current study, however, response categories were not distinct in the ‘strongly disagree’ and ‘disagree’ categories [18]. Further research is needed to test whether reducing the number of eHEALS response categories would better reflect the range of eHealth literacy levels.

Contrary to our findings, previous studies using classical test theory and item response theory (in a student population, healthy adults using the internet and patients with rheumatic disease) have supported unidimensionality of the eHEALS [17,34]. In our analysis, we identified two constructs, or concepts, of eHealth literacy: (1) items 1–5 (around knowledge about resources) and (2) items 6–8 (around evaluation of resources). A Likert-scale, such as the eHEALS, depends on unidimensionality to be able to sum its items [35]. A possible explanation for this discrepancy may be related to the age of the population studied with this study focusing on an older population (mean age: 66 years old). It could be that the knowledge to use the Internet and the ability to evaluate online resources are two distinct skills for an older population but not for a younger one that is more familiar with the electronic medium. Further research is therefore required in different populations to explore whether the eHEALS is indeed composed of two separate constructs, which may then mean summing item scores across constructs is not appropriate.

The HLQ has previously been found to have a good level of construct validity and a composite reliability of ≥0.8 for almost all subscales [14,36]. However, the scale developers had noted that participants had difficulty distinguishing between the response categories for subscales six to nine (‘very difficult’, ‘quite difficult’, ‘quite easy’ and ‘very easy’) [14]. This was confirmed in their follow-up paper using a Bayesian model approach where subscales six to eight were found to have disordered thresholds [20]. Similar findings were observed in this Rasch analyses as subscales six to nine displayed disordered thresholds. Disordered thresholds are not uncommon in scales with multiple response categories or when wording between them is similar [23,35]. If participants are not able to distinguish between response categories, the sum of the items does not truly reflect the individual’s ability i.e. selecting ‘quite’ difficult would be equivalent to selecting ‘very difficult’. The Rasch analysis demonstrated that collapsing two response categories (‘very difficult’ and ‘quite difficult’) resulted in ordered thresholds. This improved the ability for participants to distinguish between the response categories and ultimately, could enable healthcare providers to accurately measure the range of health literacy levels.

This is the first study to have assessed and compared the internal construct validity of the eHEALS and the HLQ scales using Rasch analysis in a population with CVD. Validation of such scales is particularly important in order to accurately measure the health literacy of patients with chronic disease, such as CVD, who are active participants in the management of their health. This study was able to provide new insight into the measurement properties of two commonly used scales for eHealth and health literacy by comparing prior findings. It was also able to provide information regarding item bias (ie. DIF), which has not yet been examined in the eHEALS. Furthermore, Rasch was able to analyse several psychometric aspects of the scale, such as the hierarchical structure of items, ordering of response categories, DIF and the summation of the scale, which are beyond the scope of classical test theory. We were, therefore, able to highlight aspects of both scales which require further investigation such as whether the eHEALS measures two distinct constructs of eHealth literacy and provide further support for rescoring subscales six to nine of the HLQ response categories in order to better reflect hierarchical distribution of health literacy. Further validation is now required to test these changes in a different population.

This research does have several limitations. Firstly, the cohort is mostly male and the group is known to have home Internet access, reducing generalizability of the findings. Secondly, both scales had many misfitting items and persons, which may in turn have contributed to the degree of misfit observed between the data and the Rasch measurement model. However, achieving model fit would have entailed deletion of items or persons, which was beyond the scope of this paper. Thirdly, this study did not seek to confirm whether individuals with higher eHealth or health literacy scores truly had better disease management. This is particularly noteworthy since a Dutch study found that perceived skills as measured by the eHEALS did not predict actual performance [34], although it is the only study to have done this analysis and further research is required to confirm this finding. Furthermore, we were not able to assess the external validity of both scales, which is an important area of health literacy. Finally, Rasch analysis is one among many tools for internal construct validity and the results of this study should be considered among the already existing validation work that has been done on these two scales.

## Conclusions

Electronic resources are valuable tools to access health information, however, patients must acquire the skills to effectively engage with and benefit from the plethora of resources available online. This is particularly true for CVD in which disease management and prevention depend largely on patients’ health literacy. For healthcare providers to optimally assess a patient’s level of health literacy, valid and reliable scales are essential. This study demonstrates the good psychometric properties of eHEALS and highlights that the HLQ appropriately measures its nine distinct aspects of health literacy. Further research is now needed to determine the extent to which higher eHEALS scores correctly identify those individuals with greater eHealth capacities and whether collapsing response categories in the last four subscales of the HLQ improves boundaries between response categories.

## Supporting information

S1 Table Rasch item and fit statistics for the electronic Health Literacy Scale (eHEALS) and Health Literacy Questionnaire (HLQ) scales. (DOCX)

## References

[1] Bo A, Friis K, Osborne RH, Maindal HT. National indicators of health literacy: ability to understand health information and to engage actively with healthcare providers—a population-based survey among Danish adults. BMC Public Health. 2014;14:1095 doi: 10.1186/1471-2458-14-1095 25339154 10.1186/1471-2458-14-1095 PMC4286937

[2] Watkins I, Xie B. eHealth literacy interventions for older adults: a systematic review of the literature. J Med Internet Res. 2014 11 10;16(11):e225 doi: 10.2196/jmir.3318 25386719 10.2196/jmir.3318 PMC4260003

[3] World Health Orhanisation. Health Promotion Glossary. 4th ed. Vol. 13. 349–64; 1998.

[4] Kripalani S, Gatti ME, Jacobson TA. Association of age, health literacy, and medication management strategies with cardiovascular medication adherence. Patient Educ Couns. 2010 11;81(2):177–81. doi: 10.1016/j.pec.2010.04.030 20684870 10.1016/j.pec.2010.04.030

[5] Lee TW, Lee SH, Kim HH, Kang SJ. Effective intervention strategies to improve health outcomes for cardiovascular disease patients with low health literacy skills: a systematic review. Asian Nurs Res. 2012 12;6(4):128–36. 10.1016/j.anr.2012.09.001 25031114

[6] Crengle S, Smylie J, Kelaher M, Lambert M, Reid S, Luke J, et al Cardiovascular disease medication health literacy among Indigenous peoples: design and protocol of an intervention trial in Indigenous primary care services. BMC Public Health. 2014;14:714 doi: 10.1186/1471-2458-14-714 25016481 10.1186/1471-2458-14-714 PMC4227024

[7] Chung S-Y, Nahm E-S. Testing reliability and validity of the eHealth Literacy Scale (eHEALS) for older adults recruited online. Comput Inform Nurs CIN. 2015 4;33(4):150–6. doi: 10.1097/CIN.0000000000000146 25783223 10.1097/CIN.0000000000000146 PMC4442634

[8] Tennant B, Stellefson M, Dodd V, Chaney B, Chaney D, Paige S, et al eHealth literacy and Web 2.0 health information seeking behaviors among baby boomers and older adults. J Med Internet Res. 2015 3 17;17(3):e70 doi: 10.2196/jmir.3992 25783036 10.2196/jmir.3992 PMC4381816

[9] Chesser A, Burke A, Reyes J, Rohrberg T. Navigating the digital divide: A systematic review of eHealth literacy in underserved populations in the United States. Inform Health Soc Care. 2016;41(1):1–19. doi: 10.3109/17538157.2014.948171 25710808 10.3109/17538157.2014.948171

[10] Norman CD, Skinner HA. eHealth Literacy: Essential Skills for Consumer Health in a Networked World. J Med Internet Res. 2006;8(2):e9 doi: 10.2196/jmir.8.2.e9 16867972 10.2196/jmir.8.2.e9 PMC1550701

[11] Parker RM, Baker DW, Williams MV, Nurss JR. The test of functional health literacy in adults: a new instrument for measuring patients’ literacy skills. J Gen Intern Med. 1995 10;10(10):537–41. 8576769 10.1007/BF02640361

[12] Davis TC, Crouch MA, Long SW, Jackson RH, Bates P, George RB, et al Rapid assessment of literacy levels of adult primary care patients. Fam Med. 1991 8;23(6):433–5. 1936717

[13] Barber MN, Staples M, Osborne RH, Clerehan R, Elder C, Buchbinder R. Up to a quarter of the Australian population may have suboptimal health literacy depending upon the measurement tool: results from a population-based survey. Health Promot Int. 2009 9;24(3):252–61. doi: 10.1093/heapro/dap022 19531559 10.1093/heapro/dap022

[14] Osborne RH, Batterham RW, Elsworth GR, Hawkins M, Buchbinder R. The grounded psychometric development and initial validation of the Health Literacy Questionnaire (HLQ). BMC Public Health. 2013;13:658 doi: 10.1186/1471-2458-13-658 23855504 10.1186/1471-2458-13-658 PMC3718659

[15] Norman CD, Skinner HA. eHEALS: The eHealth Literacy Scale. J Med Internet Res. 2006;8(4):e27 doi: 10.2196/jmir.8.4.e27 17213046 10.2196/jmir.8.4.e27 PMC1794004

[16] Mitsutake S, Shibata A, Ishii K, Okazaki K, Oka K. [Developing Japanese version of the eHealth Literacy Scale (eHEALS)]. Nihon Kōshū Eisei Zasshi Jpn J Public Health. 2011 5;58(5):361–71. 21905612

[17] Paramio Pérez G, Almagro BJ, Hernando Gómez Á, Aguaded Gómez JI. [Validation of the eHealth Literacy Scale (eHEALS) in Spanish University Students]. Rev Esp Salud Publica. 2015 6;89(3):329–38. doi: 10.4321/S1135-57272015000300010 26388346 10.4321/S1135-57272015000300010

[18] Nguyen J, Moorhouse M, Curbow B, Christie J, Walsh-Childers K, Islam S. Construct Validity of the eHealth Literacy Scale (eHEALS) Among Two Adult Populations: A Rasch Analysis. JMIR Public Health Surveill. 2016 5 20;2(1):e24 doi: 10.2196/publichealth.4967 27244771 10.2196/publichealth.4967 PMC4909391

[19] Paige SR, Krieger JL, Stellefson M, Alber JM. eHealth literacy in chronic disease patients: An item response theory analysis of the eHealth literacy scale (eHEALS). Patient Educ Couns [Internet]. 2016 9 [cited 2016 Dec 6]; Available from: http://linkinghub.elsevier.com/retrieve/pii/S0738399116304189 10.1016/j.pec.2016.09.008 PMC5538024 27658660

[20] Elsworth GR, Beauchamp A, Osborne RH. Measuring health literacy in community agencies: a Bayesian study of the factor structure and measurement invariance of the health literacy questionnaire (HLQ). BMC Health Serv Res [Internet]. 2016 12 [cited 2016 Dec 12];16(1). Available from: http://bmchealthservres.biomedcentral.com/articles/10.1186/s12913-016-1754-2 10.1186/s12913-016-1754-2 PMC5034518 27659559

[21] Tennant A, Conaghan PG. The Rasch measurement model in rheumatology: what is it and why use it? When should it be applied, and what should one look for in a Rasch paper? Arthritis Rheum. 2007 12 15;57(8):1358–62. doi: 10.1002/art.23108 18050173 10.1002/art.23108

[22] Prieto L, Alonso J, Lamarca R. Classical Test Theory versus Rasch analysis for quality of life questionnaire reduction. Health Qual Life Outcomes. 2003;1:27 doi: 10.1186/1477-7525-1-27 12952544 10.1186/1477-7525-1-27 PMC194220

[23] Pallant JF, Tennant A. An introduction to the Rasch measurement model: an example using the Hospital Anxiety and Depression Scale (HADS). Br J Clin Psychol Br Psychol Soc. 2007 3;46(Pt 1):1–18. 10.1348/014466506x96931 17472198

[24] Oates DJ, Paasche-Orlow MK. Health literacy: communication strategies to improve patient comprehension of cardiovascular health. Circulation. 2009 2 24;119(7):1049–51. doi: 10.1161/CIRCULATIONAHA.108.818468 19237675 10.1161/CIRCULATIONAHA.108.818468

[25] Redfern J, Usherwood T, Harris MF, Rodgers A, Hayman N, Panaretto K, et al A randomised controlled trial of a consumer-focused e-health strategy for cardiovascular risk management in primary care: the Consumer Navigation of Electronic Cardiovascular Tools (CONNECT) study protocol. BMJ Open. 2014 2;4(2):e004523 doi: 10.1136/bmjopen-2013-004523 24486732 10.1136/bmjopen-2013-004523 PMC3918991

[26] Collins SA, Currie LM, Bakken S, Vawdrey DK, Stone PW. Health literacy screening instruments for eHealth applications: A systematic review. J Biomed Inform. 2012 6;45(3):598–607. doi: 10.1016/j.jbi.2012.04.001 22521719 10.1016/j.jbi.2012.04.001 PMC3371171

[27] Noblin AM, Wan TTH, Fottler M. The impact of health literacy on a patient’s decision to adopt a personal health record. Perspect Health Inf Manag. 2012;9:1–13. PMC3510648 23209454

[28] Kayser L, Hansen-Nord NS, Osborne RH, Tjønneland A, Hansen RD. Responses and relationship dynamics of men and their spouses during active surveillance for prostate cancer: health literacy as an inquiry framework. BMC Public Health [Internet]. 2015 12 [cited 2016 Oct 25];15(1). Available from: http://www.biomedcentral.com/1471-2458/15/741 10.1186/s12889-015-2068-8 PMC4522061 26231177

[29] Linacre, John M. Sample Size and Item Calibration [or Person Measure] Stability [Internet]. 1994. Available from: https://www.rasch.org/rmt/rmt74m.htm

[30] Smith EV. Detecting and evaluating the impact of multidimensionality using item fit statistics and principal component analysis of residuals. J Appl Meas. 2002;3(2):205–31. 12011501

[31] Ramp M, Khan F, Misajon RA, Pallant JF. Rasch analysis of the Multiple Sclerosis Impact Scale MSIS-29. Health Qual Life Outcomes. 2009 6 22;7:58 doi: 10.1186/1477-7525-7-58 19545445 10.1186/1477-7525-7-58 PMC2706812

[32] Keith Timothy Z. Multiple Regression and Beyond. 1st ed. Boston: MA: Pearson; 2006.

[33] Shea TL, Tennant A, Pallant JF. Rasch model analysis of the Depression, Anxiety and Stress Scales (DASS). BMC Psychiatry. 2009 5 9;9:21 doi: 10.1186/1471-244X-9-21 19426512 10.1186/1471-244X-9-21 PMC2689214

[34] van der Vaart R, van Deursen AJ, Drossaert CH, Taal E, van Dijk JA, van de Laar MA. Does the eHealth Literacy Scale (eHEALS) Measure What it Intends to Measure? Validation of a Dutch Version of the eHEALS in Two Adult Populations. J Med Internet Res. 2011 11 9;13(4):e86 doi: 10.2196/jmir.1840 22071338 10.2196/jmir.1840 PMC3222202

[35] Gothwal VK, Bharani S, Reddy SP. Measuring coping in parents of children with disabilities: a rasch model approach. PloS One. 2015;10(3):e0118189 doi: 10.1371/journal.pone.0118189 25730331 10.1371/journal.pone.0118189 PMC4346261

[36] Batterham RW, Buchbinder R, Beauchamp A, Dodson S, Elsworth GR, Osborne RH. The OPtimising HEalth LIterAcy (Ophelia) process: study protocol for using health literacy profiling and community engagement to create and implement health reform. BMC Public Health. 2014;14:694 doi: 10.1186/1471-2458-14-694 25002024 10.1186/1471-2458-14-694 PMC4105165

