# Supplementary material for: Examination of an eHealth literacy scale and a health literacy scale in a population with moderate to high cardiovascular risk: Rasch analyses
Source: PLoS One. 2017 Apr 27;12(4):e0175372. doi: 10.1371/journal.pone.0175372 (PMC5407817; doi:10.1371/journal.pone.0175372)
Supplement: S1 Table — (DOCX) [file pone.0175372.s001.docx]

**S1 Appendix:** Rasch item and fit statistics for the electronic Health Literacy Scale (eHEALS) and Health Literacy Questionnaire (HLQ) scales

| **Scale/Subscale** | **Item** | **Item Statistics^ϯ^ Location (SE)** | **Item Fit Residuals*** |
| --- | --- | --- | --- |
| ***Ideal*** | *n* |  | *<±2.5* |
| **eHEALS** | 1 | 0.76 (0.08) | 2.46 |
|  | 2 | 0.41 (0.08) | -2.02 |
|  | 3 | -0.17 (0.08) | **-3.92** |
|  | 4 | -0.50 (0.08) | -2.31 |
|  | 5 | -0.18 (0.08) | -2.41 |
|  | 6 | -0.37 (0.08) | 1.13 |
|  | 7 | -0.01 (0.08) | 0.24 |
|  | 8 | 0.05 (0.08) | 1.63 |
| **HLQ Subscale-1** | 2 | 0.50 (0.15) | **-2.68** |
|  | 8 | 0.43 (0.15) | **-3.77** |
|  | 17 | 0.01 (0.15) | -2.21 |
|  | 22 | -0.94 (0.17) | **-2.52** |
| **HLQ Subscale-2** | 1 | -0.86 (0.12) | -0.30 |
|  | 10 | -0.15 (0.12) | -2.29 |
|  | 14 | 0.70 (0.12) | **-3.62** |
|  | 23 | 0.31 (0.11) | **-4.22** |
| **HLQ Subscale-3** | 6 | 0.62 (0.10) | 1.48 |
|  | 9 | -0.15 (0.11) | **-3.20** |
|  | 13 | -0.5 (0.11) | **-4.48** |
|  | 18 | -0.06 (0.11) | **-3.00** |
|  | 31 | -0.36 (0.11) | **-3.70** |
| **HLQ Subscale-4** | 3 | 0.20 (0.11) | -0.91 |
|  | 5 | 0.87 (0.11) | 0.06 |
|  | 11 | 0.03 (0.11) | **-4.94** |
|  | 15 | 0.58 (0.10) | -1.34 |
|  | 19 | -1.68 (0.12) | **-4.24** |
| **HLQ Subscale-5** | 4 | 0.67 (0.09) | **-2.58** |
|  | 7 | 0.30 (0.10) | -0.50 |
|  | 12 | 0.28 (0.10) | -2.01 |
|  | 16 | -0.34 (0.10) | 0.22 |
|  | 20 | -0.61 (0.10) | 0.43 |
| **HLQ Subscale-6** | 25 | 1.19 (0.13) | -1.78 |
|  | 27 | -1.03 (0.14) | **-3.54** |
|  | 30 | 0.09 (0.12) | **-3.05** |
|  | 38 | -0.51 (0.13) | **-3.08** |
|  | 43 | 0.29 (0.12) | **-3.84** |
| **HLQ Subscale-7** | 24 | 0.09 (0.11) | -1.78 |
|  | 31 | -1.49 (0.12) | -1.13 |
|  | 34 | 0.18 (0.12) | **-2.59** |
|  | 36 | 0.19 (0.12) | **-5.90** |
|  | 39 | 0.19 (0.11) | -0.61 |
|  | 42 | 1.22 (0.10) | 1.71 |
| **HLQ Subscale-8** | 26 | 0.18 (0.11) | **-2.57** |
|  | 29 | 0.82 (0.09) | -2.01 |
|  | 33 | -0.61 (0.12) | **-2.88** |
|  | 37 | -1.10 (0.11) | -1.91 |
|  | 41 | 0.70 (0.10) | **-3.44** |
| **HLQ Subscale-9** | 28 | -0.45 (0.11) | -1.33 |
|  | 32 | -0.31 (0.11) | -1.87 |
|  | 35 | 0.42 (0.11) | **-3.53** |
|  | 40 | -0.17 (0.11) | **-2.55** |
|  | 44 | 0.51 (0.11) | **-3.61** |

^ϯ^Expressed in linear log-odds units (logits), with mean item location set at 0 for each scale.

*Log residuals summarise the deviation of observed from expected responses. Deviations from the recommended range of +2.5, indicating item misfit, are in bold typeface^21^.
